# A review of economic evaluations of health care for people at risk of psychosis and for first-episode psychosis

**DOI:** 10.1186/s12888-022-03769-7

**Published:** 2022-02-17

**Authors:** Gemma E. Shields, Deborah Buck, Filippo Varese, Alison R. Yung, Andrew Thompson, Nusrat Husain, Matthew R. Broome, Rachel Upthegrove, Rory Byrne, Linda M. Davies

**Affiliations:** 1 Manchester Centre for Health Economics, Division of Population Health, School of Health Sciences, University of Manchester, Manchester, UK; 2 Institute of Education, University College London, London, UK; 3 Division of Psychology and Mental Health, School of Health Sciences, University of Manchester, Manchester, UK; 4 Greater Manchester Mental Health NHS Foundation Trust, Manchester, UK; 5 Centre for Youth Mental Health, University of Melbourne, Melbourne, Australia; 6 Institute for Mental and Physical Health and Clinical Translation, School of Medicine, Deakin University, Geelong, Australia; 7 Orygen, The Centre of Excellence in Youth Mental Health, University of Melbourne, Melbourne, Australia; 8 Division of Mental Health and Wellbeing, University of Warwick, Coventry, UK; 9 Institute for Mental Health, University of Birmingham, Birmingham, UK; 10 Birmingham Early Intervention Service, Birmingham Women’s and Children’s NHS Foundation Trust, Birmingham, UK

**Keywords:** Psychosis, Cost-effectiveness, Cost-utility, Economic evaluation, Systematic review

## Abstract

**Background:**

Preventing psychotic disorders and effective treatment in first-episode psychosis are key priorities for the National Institute for Health and Care Excellence. This review assessed the evidence base for the cost-effectiveness of health and social care interventions for people at risk of psychosis and for first-episode psychosis.

**Methods:**

Electronic searches were conducted using the PsycINFO, MEDLINE and Embase databases to identify relevant published full economic evaluations published before August 2020. Full-text English-language studies reporting a full economic evaluation of a health or social care intervention aiming to reduce or prevent symptoms in people at risk of psychosis or experiencing first-episode psychosis were included. Screening, data extraction, and critical appraisal were performed using pre-specified criteria and forms based on the NHS Economic Evaluation Database (EED) handbook and Consolidated Health Economic Evaluation Reporting Standards (CHEERS) checklist for economic evaluations. The protocol was registered on the PROSPERO database (CRD42018108226). Results were summarised qualitatively.

**Results:**

Searching identified 1,628 citations (1,326 following the removal of duplications). After two stages of screening 14 studies met the inclusion criteria and were included in the review. Interventions were varied and included multidisciplinary care, antipsychotic medication, psychological therapy, and assertive outreach. Evidence was limited in the at-risk group with only four identified studies, though all interventions were found to be cost-effective with a high probability (> 80%). A more substantial evidence base was identified for first-episode psychosis (11 studies), with a focus on early intervention (7/11 studies) which again had positive conclusions though with greater uncertainty.

**Conclusions:**

Study findings generally concluded interventions were cost-effective. The evidence for the population who are at-risk of psychosis was limited, and though there were more studies for the population with first-episode psychosis, limitations of the evidence base (including generalisability and heterogeneity across the methods used) affect the certainty of conclusions.

**Supplementary Information:**

The online version contains supplementary material available at 10.1186/s12888-022-03769-7.

## Background

Psychotic disorders are severe mental illnesses in which an individual’s behaviour, mood, perception and thoughts are altered [1]. An analysis of prevalence estimates for psychotic disorders estimated that the global lifetime prevalence is 7.49 per 1000 [2]. Symptoms are typically divided into positive symptoms (including hallucinations and delusions) and negative symptoms (such as withdrawal, depression and apathy).

Individuals who are in an “At-Risk Mental State” (ARMS) are at high risk of psychosis, but the development of psychotic disorder is not inevitable, making this period important for prevention [3]. Only a minority of people in an ARMS will develop psychosis; evidence suggests approximately 15–22% of people develop psychosis within a year from ARMS assessment [4, 5]. People who are experiencing first-episode psychosis have variable outcomes; some patients may experience a full recovery, others may require life-long treatment [6]. Preventing and effectively treating psychosis is important to improve the health of the population. People experiencing first-episode psychosis (FEP) have a higher mortality rate compared to the general population [7]. Over their lifetime people with psychotic disorders die around 10 to 15 years earlier when compared with the general population [7]. There is also a substantial morbidity burden. People with psychosis experience co-morbid physical and mental health problems, cognitive impairment, social exclusion (stigma and discrimination), side effects from treatment and reduced opportunities related to work and education [8–14]. The caregiver burden is also substantial, with carers reporting social isolation, psychological distress and reduced quality of life [15].

In the UK, guidelines from the National Institute for Health and Care Excellence (NICE) recommend that specialised services are available to everyone who is experiencing a FEP [1]. There is also now a focus on early detection of individuals at risk of developing FEP, with criteria available to help identify people in an ARMS [16–18]. NHS England has targets that include timely assessments and access to care for individuals with an ARMS and FEP. Both NICE and NHS England recommend that these services should be specialised Early Intervention in Psychosis (EIP) services, that is, community-based multidisciplinary teams that (1) seek to reduce the amount of time between the onset of symptoms and the start of treatment (the ‘duration of untreated psychosis’) and (2) provide comprehensive treatment that aims to promote recovery and minimise disability [19]. The duration of untreated psychosis has been correlated with poorer outcomes, highlighting the need for treatments to effectively identify and treat FEP in order to improve health in this population [20].

Meta-analysis demonstrates that early intervention services are effective using a range of outcomes (e.g., symptom severity, hospitalisation, school and work involvement) [21]. Furthermore, work from The King’s Fund highlighted that early intervention services may help to realise cost savings (largely attributed to inpatient costs) and early detection/intervention services for psychosis could reduce the need for services later on which again may be associated with a cost saving [22].

With rising healthcare costs, constrained budgets and a growth in the number and type of interventions, economic evaluations are needed to assess costs and outcomes associated with different options and support decision making with information on the value of interventions. Subsequently, the aim of this review was to examine and synthesise full economic evaluations of interventions for people at risk of psychosis and for FEP. The primary objective was to assess whether existing interventions are cost-effective. The secondary objective was to review the robustness of the evidence base through critical appraisal.

## Methods

The systematic review protocol was published on the online PROSPERO international register of systematic reviews (CRD42018108226) [23]. The research aimed to answer the following questions. What are the costs, health benefits and incremental cost effectiveness estimates of included studies of interventions for people at risk of psychosis and for FEP? How robust are the study designs, data and analysis methods of the included studies?

### Searches

Electronic searches were conducted in June 2019 and updated in August 2020 using the PsycINFO, MEDLINE and Embase databases via Ovid. Search terms included terms specific to economic evaluation and the population of interest (ARMS and FEP). Economic evaluation search terms were taken from the NHS Economic Evaluation Database (EED) published strategies [24]. Search terms for the population included psychosis, first-episode and at-risk. Free-text and standardised (MESH) subject terms were used. Search terms varied according to the database design. A pilot test of strategies was undertaken to check that all citations already known to the authors were identified. The full search strategies are provided in the supplementary material.

### Inclusion and exclusion criteria

Pre-specified inclusion and exclusion criteria were used to assess the relevance of identified articles. Inclusion criteria were (1) studies reporting a full economic evaluation (synthesising costs and health benefits), (2) studies focused on people at-risk of psychosis or experiencing FEP (with no restriction by study age/publication date), (3) studies focusing on any type of health or social care intervention aimed at preventing or reducing symptoms, (4) the comparator included could be no intervention (usual care) or an active intervention. Publications needed to be original full-text articles published in English reporting results (i.e., systematic reviews, conference abstracts and protocols were excluded). Studies not meeting these criteria were excluded during the screening process.

### Screening

Two stages of independent screening were performed: firstly, of titles and abstracts and secondly of the full papers. Two reviewers completed screening of all citations, with a third reviewer to resolve disagreements.

### Data extraction and quality appraisal

Data extraction and critical appraisal were performed using pre-specified criteria and forms based on the NHS EED handbook and Consolidated Health Economic Evaluation Reporting Standards (CHEERS) checklist for economic evaluations [25, 26]. The data extraction form is included in the supplementary material. Extracted data included information on study design, methodology, results, limitations, and risk of bias. One reviewer completed data extraction with 20% of data extraction checked by a second reviewer.

### Synthesis

Review findings are presented via narrative synthesis. It is typical for economic evaluations to be highly heterogeneous and therefore any quantitative synthesis (e.g., meta-analysis) that could be attempted would likely be uncertain and precarious [27]. Key aspects of the study design and results of included papers are summarised in tables.

## Results

**Fig. 1:**
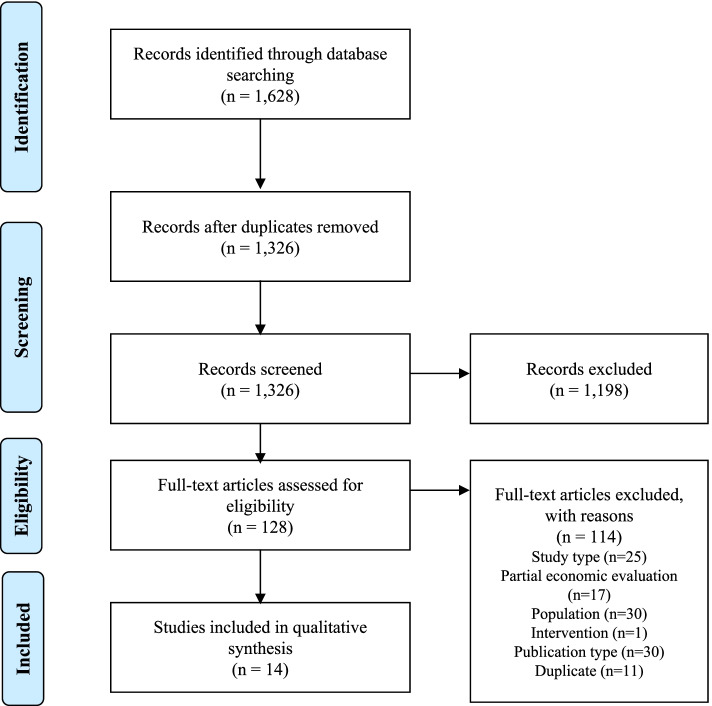
PRISMA flow diagram

Database searches identified 1,628 individual citations, following screening 14 met the inclusion criteria and were included in this review (Fig. 1).

Key characteristics of included studies are reported in Table 1.

**Table 1:** Overview of included studies

Author (year)	Population	Study design (and sample if applicable)	Setting	Intervention	Comparator	Time horizon
**At-risk populations**						
Ising et al., 2017 [28]	Ultra-high risk of psychosis	RCT •*N* = 196 •Mean age: 23 Proportion male: 51% (intervention) and 49% (comparator)	Secondary care in the Netherlands	CBT (for ultra-high risk)	Routine care	4 years
Jin et al., 2020 [29]	Clinical high-risk of psychosis	Discrete event simulation whole-disease model	Secondary care in the UK	CBT plus practice as usual	Practice as usual	Lifetime
Perez et al., 2015 [30]	General practice referrals to early intervention services	Decision tree model	Primary and secondary care in the UK	Low intensity intervention (a postal campaign consisting of biannual guidelines to help and refer individuals with early signs of psychosis) High intensity (inclusion of a specialist mental health professional who liaised with each practice and a theory-based education package)	Practice as usual	2 years
Wijnen et al., 2020 [31] ^b^	Individuals at ultra-high risk of developing psychosis (or with first-episode psychosis)	State transition (Markov) model	Secondary care in the Netherlands	Cognitive behaviour therapy	Care as usual	5-years
**First episode psychosis populations**						
Behan et al., 2020 [32]	First-episode psychosis	Retrospective cohort •*N* = 201 •Median age: 32 •Proportion male: 56%	Community care in Ireland	Early intervention (including CBT, family education and intervention, and psychosocial intervention focusing on vocational or educational needs)	Treatment as usual	1 year
Breitborde et al., 2009 [33]	First-episode psychosis	Simulation model	Community care in the USA	Multifamily group psychoeducation	Pharmacotherapy	2-, 5-, 10- and 20-year scenarios
Cocchi et al., 2011 [34]	First-episode psychosis	Retrospective cohort •*N* = 46 •Mean age: 25 (intervention) and 26 (comparator) •Proportion male: 70% (intervention) and 74% (comparator)	Secondary care in Italy	Early intervention programme (including individual pharmacotherapy, CBT, psychoeducation, motivational sessions, support group and various social group activities)	Standard care	5 years
Hastrup et al., 2013 [35]	First-episode psychosis (in contact with services for the first time)	RCT •*N* = 547 •Mean age: NR •Proportion male: NR	Secondary and community care in Denmark	Early interventions for first-episode psychosis (including assertive community treatment, psychoeducational family treatment, social skills training and low dose antipsychotic medication) for two years	Standard care (community mental health centres)	5 years
Health Quality Ontario 2018 [36]	Newly diagnosed psychosis	State transition (Markov) model	Canada	CBT for psychosis (delivered by physicians or non-physicians) plus usual care	Usual care (medications, inpatient and outpatient mental health services)	5 years
Jin et al., 2020 [29]	First-episode psychosis	Discrete event simulation whole-disease model	Secondary care in the UK	First-line oral antipsychotic medication (quetiapine, haloperidol, ariprazole, risperidone, amisulpride, olanzapine and placebo)	Interventions were compared with each other	Lifetime
				Antipsychotic medication plus family intervention	Family intervention alone or antipsychotic medication alone	
McCrone et al., 2010 [37]	First-episode psychosis or had previously disengaged without treatment	RCT •*N* = 144 •Mean age: 26 (intervention) and 27 (comparator) •Proportion male: 55% (intervention) and 74% (comparator)	Secondary and community care in the UK	Early intervention service (assertive outreach) which included low-dose medication regimes, CBT, family therapy and vocational rehabilitation	Standard care (community mental health teams with no extra training on dealing with psychosis)	18 months
Mihalopoulos et al., 2009 [38]	First-episode psychosis	Cohort with historical control group •*N* = 65 •Mean age: 22 •Proportion male: 65%	Secondary and community care in Australia	Early Psychosis Prevention and Intervention Centre (EPPIC) care (including assessment team, inpatient unit, outpatient management service and smaller therapeutic programs)	Treatment as usual (community care)	8 years
Rosenheck et al., 2016 [39]	First-episode psychosis	RCT •*N* = 404 •Mean age: 23 •Proportion male: 77% (intervention) and 66% (comparator)	Community care in the USA	Navigate early intervention package (including personalised medication management, family psychoeducation, individual resilience-focused illness self-management therapy and supported education and employment)	Standard (community) care	2 years
Stant et al., 2007 [40]	First-episode non-affective psychosis	RCT •*N* = 128 •Mean age: NR •Proportion male: 69% (intervention) and 70% (comparator)	Community care in the Netherlands	Guided discontinuation strategy (consisting of gradually tapering antipsychotic doses and eventually discontinuing antipsychotics if feasible)	Maintenance treatment	2 years
Wong et al., 2011 [41]	First-episode psychosis	Retrospective with historical control •*N* = 130 •Mean age:23 (intervention) and 24 (comparator) •Proportion male: 52% (intervention) and 54% (comparator)	Secondary and community care in Hong Kong	EASY, a specialized multi-disciplinary service programme (including public education facilitating early detection and a comprehensive intervention)	Standard care (‘pre-EASY’)—a publicly funded general psychiatric service with inpatient and outpatient service and community support	2 years

*ARMS* at-risk mental state, *CBT* cognitive behavioural therapy, *EASY* Early Assessment Service for Young People with Early Psychosis, *FEP* first episode psychosis, *RCT* randomised controlled trial

^**a**^Jin et al. (2020) [29] constructed a whole-disease model which addresses multiple decision populations and includes nineteen interventions and comparators. It is included in the table twice as it covered decision problems in the FEP population and for those at clinical high risk of psychosis

^**b**^Wijnen et al. (2020) [31] report the development of an economic model that can be used for people at high-risk and for FEP and present an example using the evidence from Ising et al. (2017) [28] in the high-risk population

### Study designs and critical appraisal

#### Population and sample

The populations considered by studies are reported in Table 1 One challenge that affects the populations considered in this review is the use of different definitions. First-episode definitions may be focused on number of service contacts, duration of psychosis or duration of antipsychotic use [42]. Duration of illness has been linked to symptoms evolving over time and the likely effect of treatment, meaning the use of varying definitions and eligibility criteria across studies will affect the results of economic evaluations [20, 43, 44].

The level of detail presented about the study samples differed; though the majority of trial papers reported the inclusion and exclusion criteria well [28, 34, 37–39]. Many of the studies had separately published papers, additional information around aspects of the study design (including the precise population) may be reported elsewhere. Common exclusion criteria included drug dependency or substance-induced psychotic disorder, and non-English language speakers [34, 37–39]. Some studies excluded people with comorbid health conditions (e.g. bipolar disorder, autism spectrum disorder and epilepsy) which may limit generalisability to broader populations [38, 39]. Typically, study participants (when reported) were young, with the mean age < 30, and the majority were male. Two studies discussed statistically significant differences between groups at baseline, which may bias results if they are associated with the cost or health benefit measure and not accounted for [32, 39]. Behan et al., compared two incidence-based cohorts and discussed that the early intervention cohort consisted of two urban and one predominantly rural catchment area, with the treatment as usual cohort from two predominantly rural catchment areas [32]. The authors noted this as a limitation of the study, but it is not clear how it may have affected results.

#### Intervention and comparator

In at-risk populations the majority of studies focused on cognitive behavioural therapy (CBT) in comparison to usual care for populations at high-risk of psychosis [28, 29, 31]. Jin et al., developed a whole-disease model which included a wide range of interventions and comparators (including CBT, antipsychotics, care settings and family interventions) [29]. Perez et al., focused on liaison approaches between primary and secondary care early intervention services for the improved detection and referral of young people at high-risk of developing psychosis [30]. Two interventions were compared to practice as usual; a low intensity intervention consisting of a postal campaign and a high intensity intervention which included a specialist mental health professional and education package.

In the population with FEP, the intervention of focus was most commonly early intervention, the exact components of which varied (described in Table 1). CBT was included in four studies, but commonly as part of a package of care [32, 34, 36, 37]. Two studies considered pharmacological options; one looking at antipsychotics for differing durations of time and another looking at a range of antipsychotics, and antipsychotics in combination with family intervention [40, 29]. A single study focused on psychoeducation [33].

Across all populations, the comparator was frequently usual care (often described as standard care or treatment as usual). However, standard care was highly variable, both in terms of how well it was reported and in terms of design when reported. This is likely to limit external validity; variability in service design and provision was noted to impact generalisability in most studies [28, 30, 32–35, 37, 39, 40]. A minority of studies explicitly justified their choice of comparator [28, 30, 36, 37].

#### Economic evaluation analysis type and health benefit measure

As shown in Table 2, cost-effectiveness analysis (11/14) was most common. Seven studies included a cost-utility analysis. Cost-utility analyses most frequently used the EQ-5D derived utility values to calculate Quality-Adjusted Life Years (QALYs). Cost-effectiveness analyses used an abundance of different measures of health benefit, including symptom scores, outcomes (such as relapse) and averted cases of psychosis. Typically, the impact of side effects was not assessed explicitly; with the exception of three studies [29, 36, 39]. Studies conducting a trial analysis and collecting a health status measure may have implicitly captured the impact of side effects.

**Table 2:** Overview of health and cost measurement

Author (year)	Type of analysis (measure of health benefit)^a^	Cost perspective
**At-risk populations**		
Ising et al., 2017 [28]	•CEA (averted psychoses)^b^ •CUA (QALY using EQ-5D)	•Health care sector •Societal
Jin et al., 2020 [29]	CUA (QALY using multiple sources for utility)	NHS and personal social services
Perez et al., 2015 [30]	CEA (true-positive referral)	NHS and personal social services
Wijnen et al., 2020 [31]	CUA (QALYs using EQ-5D)	Health care system
**First episode psychosis populations**		
Behan et al., 2020 [32]	CEA (relapse)	•Health sector •Societal
Breitborde et al., 2009 [33]	CEA (years lived with disability)	Health care system
Cocchi et al., 2011 [34]	CEA (HoNOS)	National health service
Hastrup et al., 2013 [35]	CEA (GAF)	Public sector
Health Quality Ontario 2018 [36]	•CEA (life-year saved, relapse, hospitalisation and suicide) •CUA (QALY using EQ-5D)	•Ontario Ministry of Health and Long-Term Care •Societal
Jin et al., 2020 [29]	•CUA (QALY using multiple sources for utility)	•NHS and personal social services
McCrone et al., 2010 [37]	CEA (full vocational recovery and MANSA)	Public sector (health, social care and criminal justice)
Mihalopoulos et al., 2009 [38]	CEA (Brief Psychiatric Rating Scale – Positive Symptom subscale)	Government (mental health service sector)
Rosenheck et al., 2016 [39]	•CEA (QLS-SD) ^b^ •CUA (QALY using mapping function applied to estimate utilities from PANSS scores)	Health care system
Stant et al., 2007 [40]	CUA (QALY using EQ-5D)	Societal
Wong et al., 2011 [41]	CEA (per point improvement on PANSS)	Public (health) sector

*CEA* cost-effectiveness analysis, *CUA* cost-utility analysis, *GAF* Global Assessment of Functioning, *HoNOS* Health of the Nation Outcome Scales, *MANSA* Manchester Short Assessment of Quality of Life, *PANSS* Positive and Negative Syndrome Scale, *QALY* quality-adjusted life year, *QLS-SD* one standard deviation change on the Quality of Life scale

^**a**^If the study reported QALYs, the method to obtain utilities is reported in addition

^**b**^Specified as the primary analysis or the focus of the results

#### Perspective and chosen costs

Studies most commonly took a health sector perspective (Table 2). Intervention and inpatient costs were included by all studies. Healthcare visits were included across studies although they varied by type and description, which in part is likely to be due to variations in health care delivery across settings. For example, some studies specified care settings (e.g., outpatient visits) whereas others categorised by practitioner type (e.g., psychiatrist). It should be noted that some studies included resource use related to mental health only [28, 29, 31, 38, 41] and in others it was unclear. This is restrictive as individuals with FEP and those at-risk of psychosis experience poorer physical health which may affect healthcare service use [45–47]. Other costs considered included medication [28, 29, 32, 35–41], residential care [34, 36–38] and supported housing [35, 40]. Informal care was included by two studies when a societal perspective was taken [32, 40]. Two studies included patient out of pocket costs [28, 40] and drug and alcohol services [37, 40]. McCrone et al., included costs related to criminal justice, which were collected by another study but excluded as few participants reported these [30, 37]. Though adverse event costs were not often discussed in the studies, they may be implicitly captured in service use collection for trials and may not be relevant to all interventions. Of the studies incorporating productivity losses, there were different methods used; including the friction cost approach and human capital method [28, 32, 36, 40]. Perez et al., collected data on productivity but decided to exclude it as few participants were employed and of these, there were very few reported days missed from work [30].

Five studies collected data using self-report questionnaires which, although susceptible to recall bias, offer some advantages (such as the ability to get data that is not routinely collected) and have generally been shown to be a valid method of collecting health resource data [28, 32, 37, 39, 40, 48]. Wijnen et al., utilised evidence from the study by Ising et al., which was self-report [31]. Two studies used routine data sources but supplemented this with self-report data to gather information on a greater range of services [32, 35]. Three studies used only routine data [34, 38, 41] and three studies used secondary data sources [29, 30, 36].

Study time horizons ranged from one year to lifetime, in part due to the study design (model or cohort study).

### Risk of bias

Five studies used decision analytic modelling; using different model types and structures [29–31, 33, 36]. Breitborde et al., described a simulation model tracking patients through FEP, whether they presented at mental health services and their willingness to participate [33]. A state-transition Markov model was developed by Health Quality Ontario with patients initiating in an acute phase of FEP and subsequent states included stable (with and without complications), relapse, treatment-resistant, unstable and death [36]. Finally, Perez et al., who considered an at-risk population used a decision tree model based primarily on RCT data which focused on referral and the likelihood of true or false positives [30]. Wijnen et al., report the design of a model to examine cost-effectiveness and budget impact of interventions for the prevention of psychosis and for FEP, which is intended to be adaptable to various therapies but reported initial results using the Ising et al., trial [31]. The model (called PsyMod), a state-transition Markov model, splits health states into stages: symptoms, subclinical and clinical disorder and recovery or disability or death.

All the modelling studies reported assumptions well and transparently, but authors did not always discuss how model designs were developed or validated. The two most recently identified models reported development and validation in detail [29, 31]. Wijnen et al., developed their model taking into account clinical and health economic expert opinion, and then applied it to an example using the results from Ising et al., which the authors describe as model testing [31]. Jin et al., developed a whole-disease discrete event simulation model (simulating the disease and treatment pathways), which can be used to address varying decision problems across the whole disease pathway [29]. The authors were careful to build on the evidence base by addressing common issues in schizophrenia modelling studies identified in a review, they involved multidisciplinary stakeholders in the development and validation of the model and finally they clearly report model validation and verification processes.

Five of studies were economic evaluations integrated into RCT designs [28, 35, 37, 39, 40]. A single study reported being single-blind [35]. The remaining studies either did not report blinding or were unblinded. Given that the majority focused on complex interventions, double blinding may not have been feasible. No studies were powered for economic measures. One study justified their primary analysis outcome measure (transition to psychosis) as this was powered, whereas the QALYs considered in sensitivity analysis were not [28]. Analyses were intention-to-treat, with three studies reporting imputing missing data using varied methodology [28, 39, 40].

Four studies were non-randomised and used retrospective data, making them more susceptible to bias [32, 34, 38, 41]. Cocchi et al., conducted a retrospective analysis of data for both the intervention and control [34]. Mihalopoulos et al., and Wong et al., used historical controls, matching on factors such as age, sex and diagnosis [38, 41]. Behan et al., noted that the use of historical controls limits the relevance of studies to current practice, using two contemporaneous incidence-based cohorts [32].

There were some studies with very small samples (two studies had fewer than 100 participants) which affects the validity of the results [34, 38].

A summary of the CHEERS checklist for the reporting of economic evaluations is included in the supplementary material; only one study reported sufficient detail across the full criteria [26].

### Overview of study results

Table 3 provides an overview of study results with a focus on the primary results reported (note these reflect different study time horizons and further breakdowns of results are reported within papers). Furthermore, studies often report multiple results (e.g., taking different perspectives, sensitivity analysis), consequently anyone using this evidence base for decision making should consult the full-text articles.

**Table 3:** Overview of study results

Author (year)	Brief intervention and comparator	Incremental health benefits	Currency (price year)	Incremental cost	Incremental cost-effectiveness ratio	Probability of cost-effectiveness
**At-risk populations**						
Ising et al., 2017 [28]	CBT versus routine care	• 12% more averted psychosis (SE 0.017; *P* < 0.001) ^**a**^ • 0.164 QALYs gained	US dollars (2014)	-$5,777 (95% CI − $16,952 to $4,190)	Dominant	86% ($0 per QALY)
Jin et al., 2020 [29]	CBT plus practise as usual versus practise as usual for patients at clinical high risk of psychosis	0.00 QALYs gained	UK pounds (2016/17)	-£1,243	Dominant	95% (£20,000 per QALY)
Perez et al., 2015 [30]	High intensity intervention versus low intensity intervention versus practise as usual	• 1.1 more true positives identified per practice (high intensity versus low intensity) • 0.5 more true positives identified per practice (low intensity versus practice as usual)	UK pounds (2012)	• -£1,055 (high intensity versus low intensity) • -£2,167 (low intensity versus practice as usual))	Dominant (high intensity intervention)	46% (high intensity intervention), 41% (practise as usual) and 13% (low intensity) (£0 per additional true positive)
Wijnen et al., 2020 [31] ^b^	Cognitive behavioural therapy versus care as usual	0.06 QALYs gained	Euros (2018)	- €654	Dominant	Approximately 86% ($0 per QALY)
**First-episode psychosis populations**						
Behan et al., 2020 [32]	Early intervention versus treatment as usual	0.10 (SE 0.06) relapse avoided	Euros (2012)	− €1,681 (SE €3,247)	Dominant	77% (£0 willingness to pay)
Breitborde et al., 2009 [33]	Multifamily group psychoeducation versus pharmacotherapy	• -0.23 fewer years lived with disability at 2 years (SD 0.25)^**a**^ • -88.05 fewer years lived with disability at 20 years (SD 7.32)^**a**^	US dollars (2008)	• − $6,440 at 2 years (SD $8,885)^**a**^ • − $3,882,871 at 20 years (SD $312,628) ^**a**^	Dominant	Not reported
Cocchi et al., 2011 [34]	Early intervention programme versus standard care	4.2 decrease in HoNOS	Euros (2006)	-€3,139	Dominant	Not reported
Hastrup et al., 2013 [35]	Early interventions for first-episode psychosis versus standard care	1.19 improvement on GAF (95% CI -2.65 to 5.34)	Euros (2009)	-€25,714 (SE €14,453; 95% CI -€54,113 to €2,685; *P* < 0.110)	Dominant	• Dominant in 70% of replications • 80% (50,000 Euros per unit increase in GAF)
Health Quality Ontario 2018 [36]	CBT for psychosis delivered by a non-physician plus usual care versus usual care	• 0.0157 life years gained (95% CI -0.00 to 0.04) • 0.1159 QALYs gained (95% CI 0.1159)	Canadian dollars (2017)	$2,494 (95% CI $1,472 to $3,544) ^**a**^	• $21,520 per QALY • $158,656 per life year gained	• 100% ($50,000 per QALY)
	CBT for psychosis delivered by a physician plus usual care versus CBT delivered by a non-physician	• 0.0 life years gained • 0.0 QALYs gained		$2,976 (95% CI $2,822 to $3,129) ^**a**^	Dominated	Not reported
Jin et al., 2020 [29]	First-line oral antipsychotic medication for FEP (six active treatments and placebo included)	Olanzapine had the fewest QALYs and quetiapine had the most QALYs	UK pounds (2016/17)	Amisulpride has the lowest cost per person and placebo had the highest cost per person	Mixed	Amisulpride had the greatest probability of cost-effectiveness at 39% (£20,000 per QALY)
	Antipsychotic medication plus family intervention for FEP versus family intervention alone or antipsychotic medication alone	•0.0046 QALYs gained (versus family intervention alone) •0.0184 QALYs gained (versus antipsychotic medication alone)		•-£7,160 (versus family intervention alone) •-£356 (versus antipsychotic medication alone)	Dominant	58% (£20,000 per QALY)
McCrone et al., 2010 [37]	Early intervention service versus standard care	• 6.0 improvement on MANSA (*P* = 0.025)^**a**^ • 12% more with a vocational recovery (*P* = 0.162)	UK pounds (2003/4)	-£2,318 (95% CI –£8,128 to £3,326)	Dominant	•76% (not willing to pay anything for a full or partial recovery) •92% (not willing to pay anything for a MANSA improvement)
Mihalopoulos et al., 2009 [38]	Early Psychosis Prevention and Intervention Centre care versus treatment as usual	• -1.6 BPRS total (*P* = 0.412) • -2.8 BPRS positive symptoms (*P* = 0.007)^**a**^	Australian dollars (2000/2001)	-$48,487 (95% BI = $18 161 to $85 592; *P* < 0.01)^**a**^	Dominant	Dominant in almost 100% of the iterations
Rosenheck et al., 2016 [39]	Navigate early intervention package versus standard care	0.25 improvement on the QLS (annualised) *	US dollars (2014)	$3,674 (annualised)	$14,696 per QLS-SD	94% ($40,000 per QLS-SD)
Stant et al., 2007 [40]	Guided discontinuation strategy versus maintenance treatment	0.00 QALYs gained	Euros (2004)	-€7,154 (authors stated no difference)	Not produced	Not reported
Wong et al., 2011 [41]	Multi-disciplinary service programme versus standard care	• -0.17 PANSS positive • -3.53 PANSS negative (*P* = 0.002) ^**a**^ • + 2.95 PANSS general	Hong Kong dollars (2001/02)	- $39,910	Dominant	94.4% ($0 per reduction in psychiatric inpatient admission)

*ARMS* at-risk mental state, *BPRS* Brief Psychiatric Rating Scale, *CAARMS* comprehensive assessment of at-risk mental states, *CBT* cognitive behavioural therapy, *CEA* cost-effectiveness analysis, *CUA* cost-utility analysis, *DUP* duration of untreated psychosis, *EASY* Early Assessment Service for Young People with Early Psychosis, *EQ-5D* European Quality of Life 5 dimensions, *GAF* Global Assessment of Functioning, *HoNOS* Health of the Nation Outcome Scales, *MANSA* Manchester Short Assessment of Quality of Life, *PANSS* Positive and Negative Syndrome Scale, *QALY* quality-adjusted life year, *QLS-SD* one standard deviation change on the Quality of Life scale

^**a**^Statistical significance reported (interpreted by *p* < 0.05 or 95% CI not crossing zero)

^**b**^Wijnen et al. (2020) [31] report the development of an economic model and present an example using the evidence from Ising et al. (2017)[28]

The sections below summarise the results by population.

#### At-risk populations

Few studies were identified for the population at-risk of psychosis, which limits the evidence base and ability to draw conclusions about the potential cost-effectiveness of interventions. Three of the studies focused on CBT and had broadly similar results (intervention was dominant, and probability of cost-effectiveness was quite high) [28, 29, 31]. However, the Wijnen et al., study used inputs from the Ising et al., so this is perhaps overstating a limited evidence base [28, 31]. Perez et al., concluded that intensive intervention which aimed to improve liaison between primary and secondary care for people with early signs of psychosis was cost-effective [30]. The measure of benefit used was true-positive referral which if linked to earlier effective treatment will be likely to increase health. However, it does rely on subsequent actions.

#### First-episode psychosis populations

Interventions targeting the population with FEP were typically health improving, as shown in Table 3.

Over half of the studies reported potential cost savings from intervention, though only two studies reported cost savings as statistically significant, indicating some uncertainty. Given the increase in health and frequency of cost savings being reported, it was common for studies to state that interventions were dominant (i.e., they are health increasing and cost reducing). When interventions were associated with increased incremental costs, they were typically discussed as being cost-effective. There were two exceptions, both interventions that focused on treatment with antipsychotics; one study concluded that there were no statistically significant differences between intervention and comparator (guided discontinuation of antipsychotics versus maintenance) [40] and Jin et al., found mixed results across antipsychotics, but concluded that at a threshold of £20,000 per QALY gained amisulpride is most likely to be cost-effective (39% probability), followed by risperidone (30%) and olanzapine (0.17%) [29].

Most studies conducted sensitivity analysis, though these rarely had an impact on the main conclusions and were not often comprehensive or justified (e.g. authors assumed a reduction in a single parameter without evidence). The probability of cost-effectiveness (if reported) is included in Table 3 and overall, this indicated a high likelihood of cost-effectiveness for studies reporting favourable results. There was some investigation into subgroups. Rosenheck et al., conducted a sensitivity analysis only including participants with a low duration of untreated psychosis which reduced the incremental cost-effectiveness ratio as the effect was greater in this group (compared to participants with a high duration of untreated psychosis) [39]. Behan et al., conducted subgroup analysis and found some differences; restricting to the functional psychosis subgroup was described as highly cost-effective, restricting to a younger age group (18–35) was less likely to be cost-effective and finally including people with organic psychosis, or psychosis secondary to a general medical condition (e.g., dementia) resulted in the probability of early intervention being effective falling [32].

## Discussion

The review aimed to identify full economic evaluations of interventions for people at risk of psychosis and for FEP, to assess whether existing interventions are cost-effective and to review the robustness of the evidence base through critical appraisal. The review identified 14 full economic evaluations focused on health and social care interventions for people at-risk of psychosis and with FEP.

For the at-risk group, evidence was limited, with only four studies identified in two countries. There was heterogeneity across the populations and methods in the studies, though in general the evidence appears favourable. However, the evidence overall for the at-risk group is limited in terms of the range of interventions and countries identified, so there is a need for further research in this area.

A more substantial evidence base was identified for the population with FEP, with 11 studies across a variety of countries and considering a range of intervention designs. Often (7/11) studies focused on early intervention programmes with varied design and in all but one of the identified studies this intervention was dominant versus usual care, meaning they were health improving and cost saving [32, 34, 37–39, 41]. These findings align with a prior review of early intervention for people with psychosis, which concluded early intervention is likely to be cost-effective but that the evidence is heterogeneous and methods could be improved which limits certainty [49]. Again, for the other interventions considered results were generally positive though there were some mixed findings. Whilst most results are favourable (suggesting interventions in FEP offer value for money) some caution is needed. In particular, the issues mentioned in the critical appraisal section will affect the validity and generalisability of results. The variability of early intervention makes it difficult to judge which components and designs are most cost-effective. Furthermore, not all studies investigated uncertainty comprehensively and where a probability of cost-effectiveness was presented, it was not always high.

This review has some limitations. Grey literature and non-English language studies were outside the scope of this review. Unpublished literature may be more likely to report inconclusive or negative findings [50]. We chose to focus on full economic evaluations, which synthesise health benefits and costs to provide an assessment of value for money. Partial economic evaluations may offer some useful information for decision-makers and researchers if they are focused on health benefits or costs. Studies which included participants with a significant duration of illness were excluded, which may limit the scope of the review. These, and others in the wider population with psychosis, may be useful to decision makers and researchers considering psychosis more generally.

There are many limitations with the current evidence base. Most notably uncertainty around the results with few studies reporting significant findings, limited sensitivity analysis and heterogeneity across methods. It should be noted that a high level of heterogeneity in study objectives and methods was anticipated and is in alignment with the conclusions of previous reviews of cost-effectiveness in groups with severe mental illness [23, 49, 51]. There was considerable variation in the health benefit measure used in studies; whilst interesting and relevant to the interventions, this heterogeneity limits comparability between studies. For some measures there is no agreed threshold for the willingness to pay to gain a unit of outcome, whereas accepted thresholds exist for a QALY, making interpretation easier [52–54]. It is important to consider whether the measures used are important to people with psychosis or at high-risk of psychosis. A recent qualitative study concluded that aspects important to people at high-risk (e.g., wellbeing and resilience) are not included in conventional measures [55]. A further paper identified that, as well as symptom improvements, service users prioritised social and functional ability and satisfaction [56]. Work is ongoing in this area, including the development of new quality of life measures [57]. With shorter time horizons it is questionable whether the authors were able to capture all important differences in outcomes and costs between the interventions being compared, as is recommended in economic evaluation guidelines [58]. For early intervention services there is some evidence to suggest that longer-term provision has benefits (up to 10 years) though more research is needed [59, 60]. As treatments evolve over time, it is likely that the comparator arms used (e.g., usual care) may become outdated. E.g., early intervention services may become standard practise and therefore be reflected in treatment as usual arms. Some of the trials identified were very small (*n* < 100), and applied inclusion and exclusion criteria that may not be reflective of the broader population. Only one study comprehensively reported every item on the CHEERS statement, demonstrating a need for studies to improve reporting [29].

Several areas for future research have been identified from this review. Many of the included studies emphasised a need for studies with larger sample sizes and only two studies investigated the impact of patient characteristics on cost-effectiveness. Acknowledging patient heterogeneity may increase efficiency and result in population gains if there are differing results across subpopulations [61]. Longer-term evidence is needed to explore cost-effectiveness reflecting remission and relapse from FEP over time, and the potential for intervention to impact costs and health benefits over a longer duration. Given that current guidelines include detection and treatment of those at risk of psychosis, methods to effectively identify people at-risk should also be evaluated for clinical and cost-effectiveness [1]. More similar methods across studies would make it easier to summarise the evidence base for decision makers. For example, McCrone et al., recommended future studies used QALYs and investigate the EQ-5D versus alternative measures for utility [37]. Key benefits of QALYs are that they can be easily compared across studies and willingness to pay thresholds are known, although there is mixed evidence for the validity of generic measures, such as the EQ-5D, in the population with severe mental illness [62, 63]. The review included any health and social care interventions, but identified only one study focusing outside of mental health symptoms (on weight management), which had to be excluded as results specific to the FEP were not reported separately [64]. Given the chances of reduced physical health in this population, including increased cardiovascular disease which is linked to premature mortality, this is an important gap in the evidence base [65]. People with first-episode schizophrenia have been shown to respond well to antipsychotics and these are typically a standard treatment in this population [66]. We found very limited evidence focusing on the cost-effectiveness of antipsychotics in this population. Though there are existing reviews of the cost-effectiveness of antipsychotics these cover wider populations [66–68]. As people with FEP are likely to respond differently compared to populations with more established conditions and given the variation in cost and side effect profiles of antipsychotics, more research is needed to identify which antipsychotics are most cost-effective in this group. Stant et al., considered a strategy of tapering and discontinuing antipsychotic treatment (i.e., withdrawal of standard treatment) which may currently contradict guidelines for standard care in this population.

Finally, evidence is from a limited number of countries, with no papers from low- or middle-income countries identified, despite a high burden associated with mental ill health in these settings [69]. Three-quarters of the global burden of mental, neurological and substance use disorders lie in low and middle-income countries (LMIC), yet 90% of this population does not have access to mental health care [70], or mental health research, which builds the foundation for evidence-based person-centred care. Psychosis is one of the 20 leading causes of disability worldwide, affecting 29 million people [71] contributing to major burden in LMIC. Global health research has now started to receive due attention from funders in high income countries and there are ongoing studies in ARMS in LMIC [72]. Research covering a wider range of settings and across LMIC is needed to ensure decision makers have evidence relevant to their locality.

As noted, the review findings align with a previous review by Aceituno et al., which described largely positive findings for early intervention services for people with psychosis, and which also reported issues with the evidence base [49]. A recent study reviewed the cost-effectiveness of intervention in the group at ultra-high risk of psychosis (i.e. ARMs) and again came to similar conclusions; that whilst study results are predominantly positive there are limitations to the evidence base [73]. This is the first review to synthesise cost-effectiveness results across the initial stages of psychosis.

## Conclusions

There is a substantial health and economic burden associated with psychotic disorders and so interventions to prevent psychosis and effectively treat FEP are needed. With rising healthcare costs, constrained budgets and a growth in the number and type of interventions, economic evaluations are needed to assess the value of interventions. Whilst most studies concluded that interventions for people at risk of psychosis or experiencing FEP are cost-saving or cost-effective, results were varied. However, the review identified several gaps in the literature (e.g., a paucity of high-quality studies, evidence in ARMS and evidence in low- or middle- income countries). A key limitation is that studies were heterogeneous (e.g., using different health benefit measures), which limits comparison across studies: addressing this within the research community should be a key focus going forward.

## Supplementary Information

**Additional file 1.**

## Data Availability

The datasets used during the current study (data extraction forms) are available from the corresponding author on reasonable request.

## Abbreviations

ARMS: At-risk mental state
BPRS: Brief Psychiatric Rating Scale
CAARMS: Comprehensive assessment of at-risk mental states
CBT: Cognitive behavioural therapy
CEA: Cost-effectiveness analysis
CHEERS: Consolidated Health Economic Evaluation Reporting Standards
CUA: Cost-utility analysis
DUP: Duration of untreated psychosis
EASY: Early Assessment Service for Young People with Early Psychosis
EED: Economic Evaluation Database
EIP: Early Intervention in Psychosis
EPPIC: Early Psychosis Prevention and Intervention Centre
FEP: First episode psychosis
GAF: Global Assessment of Functioning
HoNOS: Health of the Nation Outcome Scales
LYG: Life years gained
NICE: National Institute for Health and Care Excellence
MANSA: Manchester Short Assessment of Quality of Life
PANSS: Positive and Negative Syndrome Scale
QALYS: Quality-Adjusted Life Years
QLS-SD: One standard deviation change on the Quality of Life scale
RCT: Randomised controlled trial
