# Supplementary material for: A review of economic evaluations of health care for people at risk of psychosis and for first-episode psychosis
Source: BMC Psychiatry. 2022 Feb 17;22:126. doi: 10.1186/s12888-022-03769-7 (PMC8851734; doi:10.1186/s12888-022-03769-7)
Supplement: Supplementary file 1 — Additional file 1. [file 12888_2022_3769_MOESM1_ESM.docx]

# Supplementary material

**Search strategies**

***Medline***

1 psychosis/ or psychosis.mp. or psychoses.mp.

2 exp Psychotic Disorders/ or psychotic disorders.mp. or psychotic disorder.mp.

3 1 or 2

4 first episode.mp.

5 (initial or first or recent onset or early).mp.

6 (clinically at high risk or clinically at risk or clinical high risk or ultra-high risk or prodrom* or at risk mental state or risk of psychosis or ARMS or prodromal psychosis).mp.

7 4 or 5 or 6

8 Economics/

9 exp "Costs and Cost Analysis"/

10 Value of Life/

11 Economics, Dental/

12 exp Economics, Hospital/

13 Economics, Medical/

14 Economics, Nursing/

15 Economics, Pharmaceutical/

16 8 or 9 or 10 or 11 or 12 or 13 or 14 or 15

17 (econom$ or cost or costs or costly or costing or price or prices or pricing or pharmacoeconomic$).ti,ab.

18 (expenditure$ not energy).ti,ab.

19 (value adj1 money).ti,ab.

20 budget$.ti,ab.

21 17 or 18 or 19 or 20

22 16 or 21

23 ((energy or oxygen) adj cost).ti,ab.

24 (metabolic adj cost).ti,ab.

25 ((energy or oxygen) adj expenditure).ti,ab.

26 23 or 24 or 25

27 22 not 26

28 3 and 7 and 27

29 (letter or editorial or note or news or congresses or case reports).pt.

30 28 not 29

31 limit 30 to (english language and human)

***Embase***

1 psychosis/ or psychosis.mp. or psychoses.mp.

2 exp Psychotic Disorders/ or psychotic disorders.mp. or psychotic disorder.mp.

3 1 or 2

4 first episode.mp.

5 ((initial or first or recent onset or early) adj3 (psychosis or psychotic or schizo*)).mp.

6 (clinically at high risk or clinically at risk or clinical high risk or ultra-high risk or prodrom* or at risk mental state or risk of psychosis or ARMS or prodromal psychosis).mp.

7 4 or 5 or 6

8 Health Economics/

9 exp Economic Evaluation/

10 exp Health Care Cost/

11 exp Pharmacoeconomics/

12 8 or 9 or 10 or 11

13 (econom$ or cost or costs or costly or costing or price or prices or pricing or pharmacoeconomic$).ti,ab.

14 (expenditure$ not energy).ti,ab.

15 (value adj2 money).ti,ab.

16 budget$.ti,ab.

17 13 or 14 or 15 or 16

18 12 or 17

19 letter.pt.

20 editorial.pt.

21 note.pt.

22 19 or 20 or 21

23 3 and 7 and 18

24 23 not 22

25 ((energy or oxygen) adj cost).ti,ab.

26 (metabolic adj cost).ti,ab.

27 ((energy or oxygen) adj expenditure).ti,ab.

28 25 or 26 or 27

29 24 not 28

30 limit 29 to (english language and human)

***PsychINFO***

1 psychosis/ or psychosis.mp. or psychoses.mp.

2 exp Psychotic Disorders/ or psychotic disorders.mp. or psychotic disorder.mp.

3 1 or 2

4 first episode.mp.

5 ((initial or first or recent onset or early) adj3 (psychosis or psychotic or schizo*)).mp.

6 (clinically at high risk or clinically at risk or clinical high risk or ultra-high risk or prodrom* or at risk mental state or risk of psychosis or ARMS or prodromal psychosis).mp.

7 4 or 5 or 6

8 "Costs and Cost Analysis"/

9 "Cost Containment"/

10 (economic adj2 evaluation$).ti,ab,id.

11 (economic adj2 analy$).ti,ab,id.

12 (economic adj2 (study or studies)).ti,ab,id.

13 (cost adj2 evaluation$).ti,ab,id.

14 (cost adj2 analy$).ti,ab,id.

15 (cost adj2 (study or studies)).ti,ab,id.

16 (cost adj2 effective$).ti,ab,id.

17 (cost adj2 benefit$).ti,ab,id.

18 (cost adj2 utili$).ti,ab,id.

19 (cost adj2 minimi$).ti,ab,id.

20 (cost adj2 consequence$).ti,ab,id.

21 (cost adj2 comparison$).ti,ab,id.

22 (cost adj2 identificat$).ti,ab,id.

23 (pharmacoeconomic$ or pharmaco-economic$).ti,ab,id.

24 8 or 9 or 10 or 11 or 12 or 13 or 14 or 15 or 16 or 17 or 18 or 19 or 20 or 21 or 22 or 23

25 (task adj2 cost$).ti,ab,id.

26 (switch$ adj2 cost$).ti,ab,id.

27 (metabolic adj cost).ti,ab,id.

28 ((energy or oxygen) adj cost).ti,ab,id.

29 ((energy or oxygen) adj expenditure).ti,ab,id.

30 25 or 26 or 27 or 28 or 29

31 editorial.dt.

32 letter.dt.

33 dissertation abstract.pt.

34 31 or 32 or 33

35 3 and 7 and 24

36 35 not 30

37 36 not 34

38 limit 37 to (english language and human)

**Table 1 Data extraction form**

| **Author** |  |
| --- | --- |
| **Year of publication** |  |
| **Title** |  |
| **Subject of study** | |
| **Condition** |  |
| **Intervention (s)** |  |
| **Comparator (s)** |  |
| **Population** |  |
| **Country** |  |
| **Time horizon** |  |
| **Hypothesis/study question** |  |
| **Features of study** | |
| **Type of study (model or trial)** |  |
| **Perspective used** |  |
| **Type of economic evaluation** |  |
| **Date (s) when data was collected** |  |
| **Clinical evidence** | |
| **Was clinical evidence from single study or multiple sources?** |  |
| **If single study:** | |
| **Study sample** |  |
| **Study design** |  |
| **Analysis of effectiveness** |  |
| **Effectiveness results** |  |
| **Clinical conclusions** |  |
| **If multiple sources:** | |
| **Clinical and epidemiological data used** |  |
| **Data sources** |  |
| **Methods used to obtain data** |  |
| **Measure of health benefit** | |
| **Summary measure of health benefit** |  |
| **Method of valuation of health states** |  |
| **Discount rate applied to health benefit** |  |
| **Direct costs** | |
| **Direct costs included** |  |
| **Source of data** |  |
| **Direct cost discount rate** |  |
| **Date (s) price data is applicable to** |  |
| **Were resource quantities and costs reported separately?** |  |
| **Inflation adjustment applied** |  |
| **Excluded costs** |  |
| **Currency and price year** |  |
| **Indirect costs (productivity)** |  |
| **Indirect costs included** |  |
| **Source of cost data** |  |
| **Cost and quantity reported seperately?** |  |
| **Date (s) price data is applicable to** |  |
| **Indirect costs discount rate** |  |
| **Statistical analysis of quantities/costs** |  |
| **Was the study powered to detect statistical difference?** |  |
| **Methods used to allow for uncertainty** | |
| **If modelling study:** | |
| **Was parameter uncertainty investigated and how?** |  |
| **Was structural uncertainty investigated and how?** |  |
| **Was patient heterogeneity investigated?** |  |
| **Was EVPI calculated?** |  |
| **If non-modelling study:** | |
| **Was variability in data investigated and how?** |  |
| **Results** | |
| **Estimated benefits** |  |
| **Incremental benefits of intervention** |  |
| **Incremental benefits of comparator** |  |
| **Sensitivity analysis of benefits only** |  |
| **Were side effects considered?** |  |
| **Cost results** | |
| **Total intervention cost** |  |
| **Total comparator cost** |  |
| **Incremental costs** |  |
| **Were adverse effect costs dealt with?** |  |
| **Sensitivity analysis for costs only** |  |
| **Synthesis of costs and benefits** | |
| **Method of benefit and cost combination** |  |
| **Incremental analysis** |  |
| **Willingness to pay threshold** |  |
| **Probability of cost-effectiveness** |  |
| **Summary of findings** |  |
| **Differences in cost-effectiveness of sub-populations** |  |
| **Summary of authors conclusions** |  |
| **Critical commentary** | |
| **Population and sample** |  |
| **Intervention and comparator** |  |
| **Analysis type and health benefit measure** |  |
| **Perspective and costs included** |  |
| **Resource use and price/unit costs** |  |
| **Other** |  |

**Table 2 CHEERS checklist**

Key: 0 = Not reported, 1= Partly reported, 2 = Well reported, - = Not applicable.

| **Paper ID** | **Behan et al. 2020** | **Breitborde et al 2009** | **Cocchi et al 2011** | **Hastrup et al 2013** | **Health Quality Ontario 2018** | **Ising et al 2017** | **Jin et al 2020** | **McCrone et al 2010** | **Mihalopoulos et al 2009** | **Perez et al 2015** | **Rosenheck et al 2016** | **Stant et al 2007** | **Wijnen et al 2020** | **Wong et al 2011** |
| --- | --- | --- | --- | --- | --- | --- | --- | --- | --- | --- | --- | --- | --- | --- |
| **1. Title** | 1 | 2 | 2 | 2 | 1 | 2 | 2 | 2 | 1 | 1 | 2 | 1 | 1 | 1 |
| **2. Abstract** | 1 | 1 | 1 | 1 | 1 | 1 | 2 | 1 | 1 | 1 | 1 | 1 | 1 | 1 |
| **3. Background and objectives** | 2 | 2 | 2 | 2 | 2 | 2 | 2 | 2 | 2 | 2 | 2 | 2 | 2 | 2 |
| **4. Target population and subgroups** | 2 | 1 | 2 | 2 | 2 | 2 | 2 | 2 | 2 | 1 | 2 | 2 | 1 | 2 |
| **5. Setting and location** | 2 | 2 | 2 | 2 | 2 | 2 | 2 | 2 | 2 | 2 | 2 | 2 | 1 | 2 |
| **6. Study perspective** | 2 | 0 | 2 | 2 | 2 | 2 | 2 | 2 | 2 | 1 | 2 | 2 | 2 | 2 |
| **7. Comparators** | 2 | 2 | 2 | 2 | 2 | 2 | 2 | 2 | 2 | 2 | 2 | 2 | 2 | 2 |
| **8. Time horizon** | 2 | 2 | 2 | 2 | 2 | 2 | 2 | 1 | 2 | 2 | 1 | 2 | 2 | 2 |
| **9. Discount rate** | - | 2 | 1 | 2 | 2 | 1 | 2 | 2 | 2 | 2 | 0 | - | 2 | 1 |
| **10. Choice of health outcomes** | 2 | 2 | 2 | 1 | 2 | 2 | 2 | 1 | 1 | 2 | 2 | 1 | 1 | 1 |
| **11a. Measurement of effectiveness** | 1 | - | 1 | 1 | - | 1 | - | 1 | 1 | - | 1 | 1 | - | 1 |
| **11b. Measurement of effectiveness** | - | 2 | - | - | 2 | - | 2 | - | - | 2 | - | - | 1 | - |
| **12. Measurement and valuation of preference-based outcomes** | - | - | - | - | 1 | 2 | 2 | - | - | - | 2 | 2 | 1 | - |
| **13a. Estimating resources and costs** | 2 | - | 2 | 2 | - | 2 | - | 2 | 2 | - | 2 | 2 | - | 2 |
| **13b. Estimating resources and costs** | - | 2 | - | - | 2 | - | 2 | - | - | 2 | - | - | 1 | - |
| **14. Currency, price date, and conversion** | 2 | 2 | 2 | 2 | 2 | 2 | 2 | 2 | 2 | 1 | 2 | 2 | 2 | 2 |
| **15. Choice of model** | - | 1 | - | - | 2 | - | 2 | - | - | 2 | - | - | 2 | - |
| **16. Assumptions** | - | 2 | - | - | 2 | - | 2 | - | - | 2 | - | - | 2 | - |
| **17. Analytical methods** | 1 | 2 | 1 | 2 | 2 | 2 | 2 | 2 | 1 | 2 | 2 | 2 | 2 | 1 |
| **18. Study parameters** | 1 | 2 | 2 | 1 | 2 | 2 | 2 | 2 | 1 | 2 | 2 | 1 | 1 | 2 |
| **19. Incremental costs and outcomes** | 1 | 2 | 2 | 2 | 2 | 2 | 2 | 2 | 1 | 2 | 1 | 1 | 1 | 1 |
| **20a. Characterising uncertainty** | 2 | - | 1 | 2 | - | 2 | - | 2 | 2 | - | 2 | 1 | - | 1 |
| **20b. Characterising uncertainty** | - | 1 | - | - | 2 | - | 2 | - | - | 1 | - | - | 2 | - |
| **21. Characterising heterogeneity** | 2 | - | - | - | - | - | - | - | 2 | - | 1 | - | - | - |
| **22. Discussion (Study findings, limitations, generalisability, and current knowledge)** | 2 | 1 | 2 | 2 | 2 | 2 | 2 | 2 | 1 | 1 | 2 | 2 | 1 | 1 |
| **23. Source of funding** | 2 | 2 | 2 | 0 | 0 | 2 | 2 | 2 | 0 | 2 | 2 | 2 | 2 | 2 |
| **24. Conflicts of interest** | 2 | 2 | 0 | 0 | 0 | 2 | 2 | 2 | 0 | 2 | 2 | 0 | 2 | 0 |

Note that study reporting will vary according to journal requirements. Additionally, most studies were conducted alongside randomised controlled trials and therefore items not reported may have been reported elsewhere.
